# Comprehensive analysis of Syk gene methylation in colorectal cancer

**DOI:** 10.1002/iid3.449

**Published:** 2021-05-12

**Authors:** Fanqin Bu, Xiaojian Zhu, Sicheng Liu, Kang Lin, Jinfeng Zhu, Jun Huang

**Affiliations:** ^1^ Department of Gastrointestinal Surgery Second Affiliated Hospital of Nanchang University Nanchang Jiangxi Province China; ^2^ Department of Research Center Seventh Affiliated Hospital of Sun Yat‐sen University Shenzhen China

**Keywords:** c‐Myc, colorectal cancer, metastasis, Syk

## Abstract

**Background**: Metastasis of colorectal cancer (CRC) extremely affects the prognosis of CRC patients. Recently, the genetic methylation has been shown to associate with tumor metastasis. This research aimed to explore the Syk gene, which is frequently hypermethylated in different cancers, and its impact on the metastasis of CRC cells.

**Methods**: We employed the UALCAN database for the detection of the methylation levels of Syk in different cancers. CIBERSORT, TIMER and TISIDB tools were employed to analyze the association of Syk expression with immune features of CRC. Treatment with decitabine has been noted to restore the expression of Syk in CRC cells. The invasion and migration abilities of CRC cell lines were determined using transwell and wound healing assays. The correlation between Syk and c‐Myc was established using the GEPIA2 database and Western blot assays.

​**Results**: Our results, based on UALCAN, revealed that the methylation level of Syk was altered in diverse cancers including colon adenocarcinoma. We found that expression profile and methylation level of Syk was correlated with immune features of colon adenocarcinoma. Decitabine can restore the expression of Syk in HCT116 and SW480 cells, hence affecting their migration and invasion. Results from GEPIA2 showed that Syk expression was correlated with c‐Myc, while Western blotting analysis revealed a negative association between the expression level of Syk and c‐Myc.

​**Conclusions**:​ ​This study demonstrates that the expression of Syk could be restored by decitabine in colorectal cancer, thus affecting the migration and invasion abilities of CRC cells.

## Graphical abstract

In our study, we aimed to research the correlation of methylation level of Syk and metastasis of colorectal cancer (CRC). We further used Decitabine to restore the expression of Syk in CRC cells. The results revealed that Decitabine could restore the expression of Syk and therefore prohibited the migration and invasion of CRC cells.

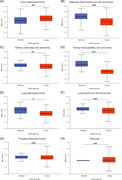

## 1 INTRODUCTION

Colorectal cancer (CRC) is one of the most prevalent malignant tumors worldwide.^1^ The risk factors for this tumor include obesity, lack of physical exercise, and smoking. Worldwide incidence and mortality of CRC are continuously rising, becoming the fourth most deadly cancer.^2^ The currently preferred treatment modality for CRC is surgery, but surgical treatment for metastatic CRC patients remains poor.^3^ According to statistics from the American Cancer Society, the 5‐year survival rate of CRC confined to the intestinal wall (Stage I and II) after surgical resection is close to 90%, and once regional lymph node (Stage III) or distant organ (Stage IV) metastasis occurs, the 5‐year survival rate drops rapidly to 70% or 12%, respectively.^4^ This suggests that tumor metastasis may be an important factor affecting the prognosis of CRC patients.

Recently, gene hypermethylation has been demonstrated to affect tumor metastasis and invasion.^5^, ^6^ In addition, genetic mutations and epigenetic changes are considered to be the root causes of tumor development, invasion, and metastasis.^7^ Chromosomal and microsatellite instabilities are important genetic bases for the development of CRC.^8^ These studies have proved the role of genetic methylation in cancer development and research prospects for genetic methylation. Demethylation drugs such as decitabine have been approved by the US FDA for the clinical treatment of myelodysplastic syndrome and leukemia.^9^ Syk is a non‐receptor tyrosine kinase that is located in chromosome 9q22.1–9q22.3, exhibiting an essential role in the signaling pathway of the receptors. It has been stated that methylation of the Syk promoter region can promote the proliferation and metastasis of CRC cells, which is directly associated with the prognosis of CRC patients.^10^ The discovery of immune checkpoints has revolutionized the application of immunotherapy in the treatment of metastatic CRC.^11^, ^12^ Studies have shown that TGFβ is elevated in the tumor microenvironment which enables tumor cells to evade immune killing. This protein promotes T cell rejection and prevents the acquisition of Type 1 T‐helper cell effector phenotype.^12^ But none has investigated the association of Syk gene with immune features. In this study, we aimed to explore how Syk gene expression level affects the invasion and migration of CRC cells. We also assessed the relationship between the expression and methylation of Syk with immune features of CRC.

## 2 MATERIALS AND METHODS

### 2.1 UALCAN

UALCAN is an effective cancer data online analysis and mining website, mainly based on the relevant cancer data from the TCGA database.^13^ We used UALCAN to compare the methylation level of Syk in different cancers and paired normal tissues. The *p *value was calculated using the student's *t*‐test. The differential methylation level of Syk in a cancer category was considered to occur if the results of *p* < .05.

### 2.2 CIBERSORT and TIMER

The CIBERSORT is used to analyze the cell composition of complex tissues.^14^ It was employed to perform Spearman correlation analysis to determine the association of Syk gene expression with the infiltration of 22 immune cells in colon adenocarcinoma and rectal adenocarcinoma. TIMER is a widely used tool to study the molecular characteristics of tumors and immune interactions.^15^ Herein, it was used to analyze the relationship between Syk expression and the contents of six immune cells.

### 2.3 TISIDB

The TISIDB is an online platform used to analyze the interaction between genes and immune features.^16^ It contains more powerful functions compared to CIBERSORT and TIMER. It can analyze the relationship between the methylation level of a gene and immune features. In this study, it was employed to assess the relationship between Syk methylation and the abundance of tumor‐infiltrating lymphocytes and immune‐stimulators in colon adenocarcinoma.

### 2.4 Cell lines

CRC cell lines, including SW480, HCT116, HT29, and LoVo were purchased from the Chinese Academy of Sciences. These cells were cultured in RPMI 1640 media, supplemented with 10% fetal bovine serum (FBS; Gibco), and incubated at 37°C and 5% CO_2_. Decitabine (NSC127716, 5AZA‐CdR) was replenished in the CRC cells following the manufacturer's instructions for the subsequent proceedings.

### 2.5 Western blot analysis

Total protein from CRC cells with or without the replenish of decitabine was extracted with RIPA lysate. The protein concentration was determined using a BCA assay. Then, protein samples were mixed with protein buffer and boiled in boiling water for 10 min. Equal amounts of proteins from CRC cell lines with or without the replenish of decitabine were separated on 10% gel electrophoresis and subsequently transferred to vinylidene fluoride film membranes. After electrophoresis, membranes were blocked with 5% skim milk powder for 2 h and incubated with primary antibody against Syk (1:2000; Abcam) and c‐Myc (1:1000; Abcam) at 4°C overnight. Thereafter, membranes were incubated with goat anti‐rabbit immunoglobulin G antibody for 90 min at room temperature. GAPDH served as the housekeeping gene. The blots were detected using ECL (Millipore) and developed on X‐ray film.

### 2.6 Transwell assay

For this experiment, the migration ability of CRC cells was determined using transwell chambers (Corning Costar). Briefly, HCT116 and SW480 cells with or without decitabine supplementation were lysed with FBS‐free media. These cells were subsequently replenished in the upper chamber, while the lower chamber served as a chemoattractant, which was filled with 10% FBS media. After 48 h of incubation, the cells in the upper chamber were carefully harvested. The migrated cells were collected in the lower chamber, fixed in methanol, and stained with 0.1% crystal violet. The number of cells was counted under an inverted microscope. We applied similar procedures to evaluate the invasion test, except the insert is pre‐coated with a matrix gel.

### 2.7 Wound‐healing assay

The ability of horizontal migration of CRC cells was evaluated using wound healing analysis. When the CRC cells were approximately 95% confluent, we extracted the media and scratched the monolayer vertically with a 200‐μl tip. The cell fragments were subsequently washed with PBS and returned to the incubator. The images were obtained at 0 and 48 h after wounding using an inverted microscope. The ability of wound healing was determined by measuring the gap size under the microscope.

### 2.8 GEPIA2

GEPIA2 (http://gepia2.cancer-pku.cn/) is an online platform that contains the RNA sequencing expression data of cancer and normal samples from TCGA and GTEx databases.^17^ This database was used to determine the correlations between Syk and c‐Myc expression in CRC. The correlation coefficient was calculated using the Spearman method.

### 2.9 Statistical analysis

All experiments were performed more than three times. Data were analyzed with SPSS software version 24.0 and GraphPad Prism version 7. A value of *p* < .05 was regarded statistically significant.

## 3 RESULTS

### 3.1 Methylation level of Syk in various cancers

**Figure 1:**
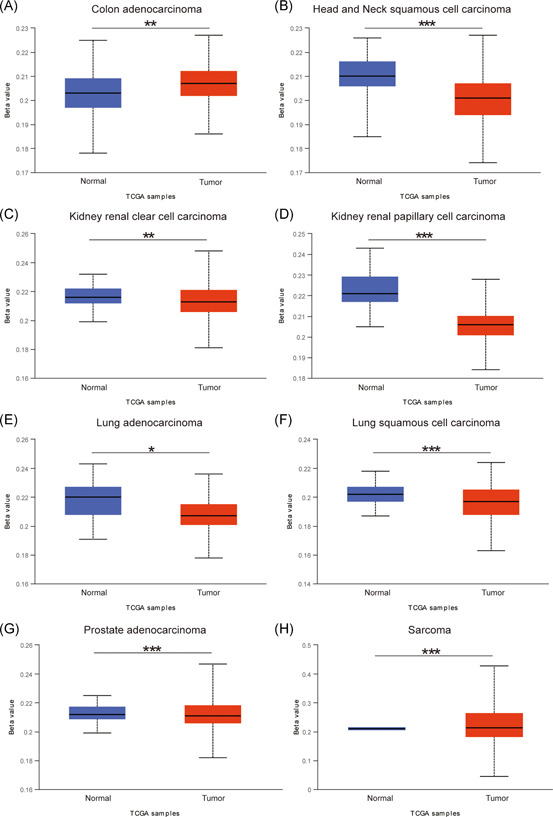
Alteration of Syk methylation levels in different cancer. (A–F) The methylation level of Syk was altered in colon adenocarcinoma, head and neck squamous cell carcinoma, kidney renal clear cell carcinoma, kidney renal papillary cell carcinoma, lung adenocarcinoma, lung squamous cell carcinoma, prostate adenocarcinoma, and sarcoma compared with paired normal tissues using UALCAN (*p < .05, **p < .01, ***p < .001)

Our results, based on the UALCAN database, revealed that the methylation level of Syk was higher in colon adenocarcinoma (Figure 1A) and sarcoma (Figure 1H) than in paired normal tissues. Conversely, the methylation level of Syk was lower in head and neck squamous cell carcinoma (Figure 1B), kidney renal clear cell carcinoma (Figure 1C), kidney renal papillary cell carcinoma (Figure 1D), lung adenocarcinoma (Figure 1E), lung squamous cell carcinoma (Figure 1F), and prostate adenocarcinoma (Figure 1G) compared with paired normal tissues.

### 3.2 Expression level of Syk correlates with immune features of colorectal cancer

**Figure 2:**
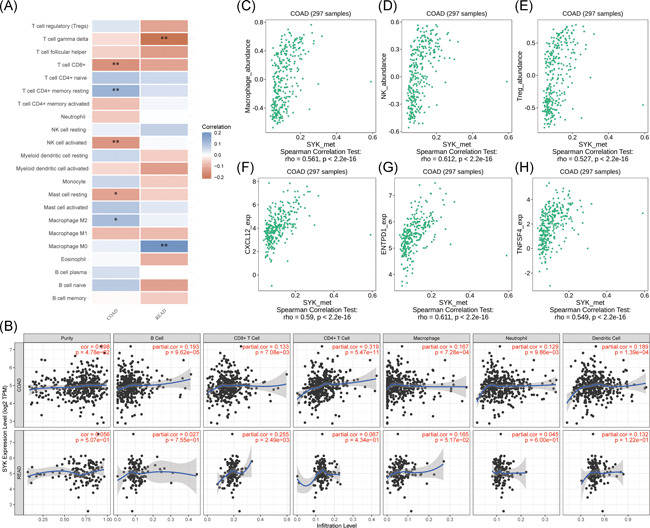
Expression and methylation level of Syk was associated with infiltration of immune cells. (A) Correlation between Syk expression and the contents of 22 immune cells in colon adenocarcinoma and rectal adenocarcinoma as determined using CIBERSORT (*p < 0.05, **p < 0.01). (B) Correlation between Syk expression and the contents of six immune cells in colon adenocarcinoma and rectal adenocarcinoma as evaluated using TIMER. (C–H) Correlation between Syk methylation and abundance of tumor‐infiltrating lymphocytes and immune‐stimulators in colon adenocarcinoma as determined using TISIDB

Studies have shown that immune infiltration influences cancer progression. Therefore, we determined the association of Syk expression with the contents of different immune cells. Results from CIBERSORT analysis revealed that the expression profile of Syk was positively correlated with CD4^+^ memory resting T cell and M2 macrophages in colon adenocarcinoma, and positively correlated with M0 macrophages in rectal adenocarcinoma (Figure 2A). Moreover, Syk expression was negatively correlated with CD8^+^ T cells, activated NK cells, and activated mast cells in colon adenocarcinoma, and negatively correlated with gamma delta T cells in rectal adenocarcinoma (Figure 2A). Results from TIMER analysis showed a tight correlation between Syk expression and infiltration of immune cells in colon adenocarcinoma (Figure 2B). In addition, the TISIDB revealed that the methylation level of Syk was strongly associated with the abundance of macrophages (Figure 2C), NK cells (Figure 2D), and Tregs (Figure 2E) in colon adenocarcinoma. The methylation level of Syk was also correlated with several immune‐stimulators such as CXCL12 (Figure 2F), ENTPD1 (Figure 2G), and TNFSF4 (Figure 2H).

### 3.3 Expression of Syk could be restored by decitabine

**Figure 3:**
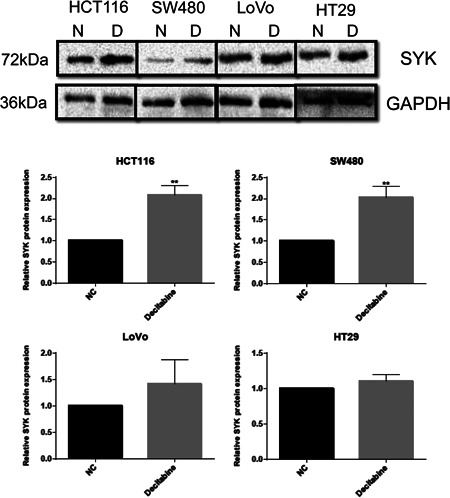
Restoration of Syk expression in HCT116 and SW480 cells by decitabine (**p < .01). D, Decitabine; N, NC

Here, we examined the effect of decitabine on Syk expression following replenishment of the CRC cells with decitabine. The Western blot analysis results revealed that the protein expression of Syk was higher in SW480 and HCT116 cells compared to those without decitabine supplement (Figure 3). In addition, the expression of Syk was not significantly changed in HT29 and LoVo cells (Figure 3). Therefore, both SW480 and HCT116 cell lines were selected for the subsequent experiments.

### 3.4 Effect of the expression of Syk on invasion and migration of CRC in vitro

**Figure 4:**
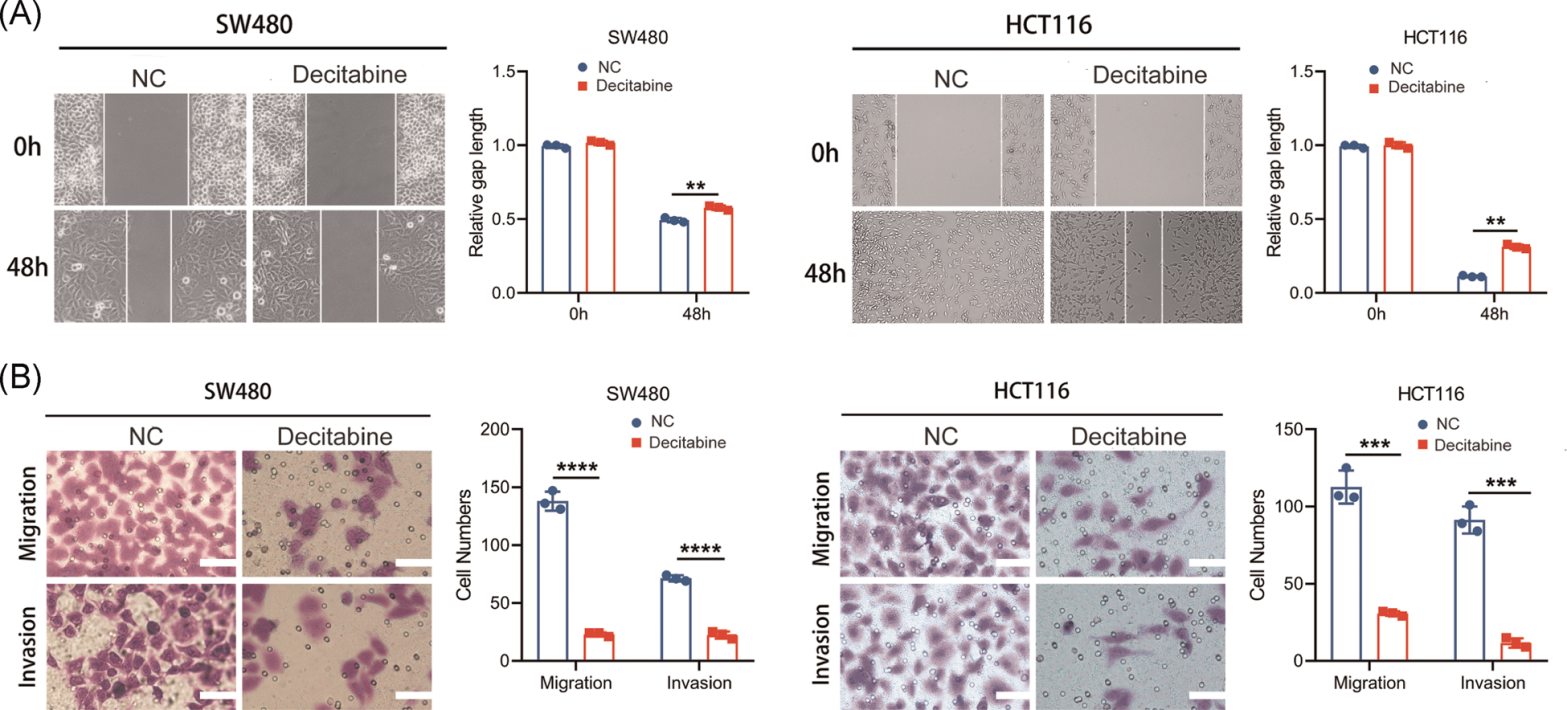
Alteration of the migration and invasion ability of CRC after replenishment with decitabine in CRC cells. (A) Wound healing assay was performed to evaluate the ability of horizontal migration of SW480 and HCT116 (**p < .01). (B) Transwell assay was performed to assess the ability of vertical migration and invasion of SW480 and HCT116. CRC, colorectal cancer. (***p < .001, ****p < .0001)

Our wound healing assays demonstrated that the horizontal migration ability of SW480 and HCT116 cells were lower after replenishment with decitabine (Figure 4A). Further, transwell assays showed that the vertical migration and invasion abilities of SW480 and HCT116 cells were lower following replenishment with decitabine (Figure 4B).

### 3.5 Regulation of c‐Myc expression in CRC cells by Syk

**Figure 5:**
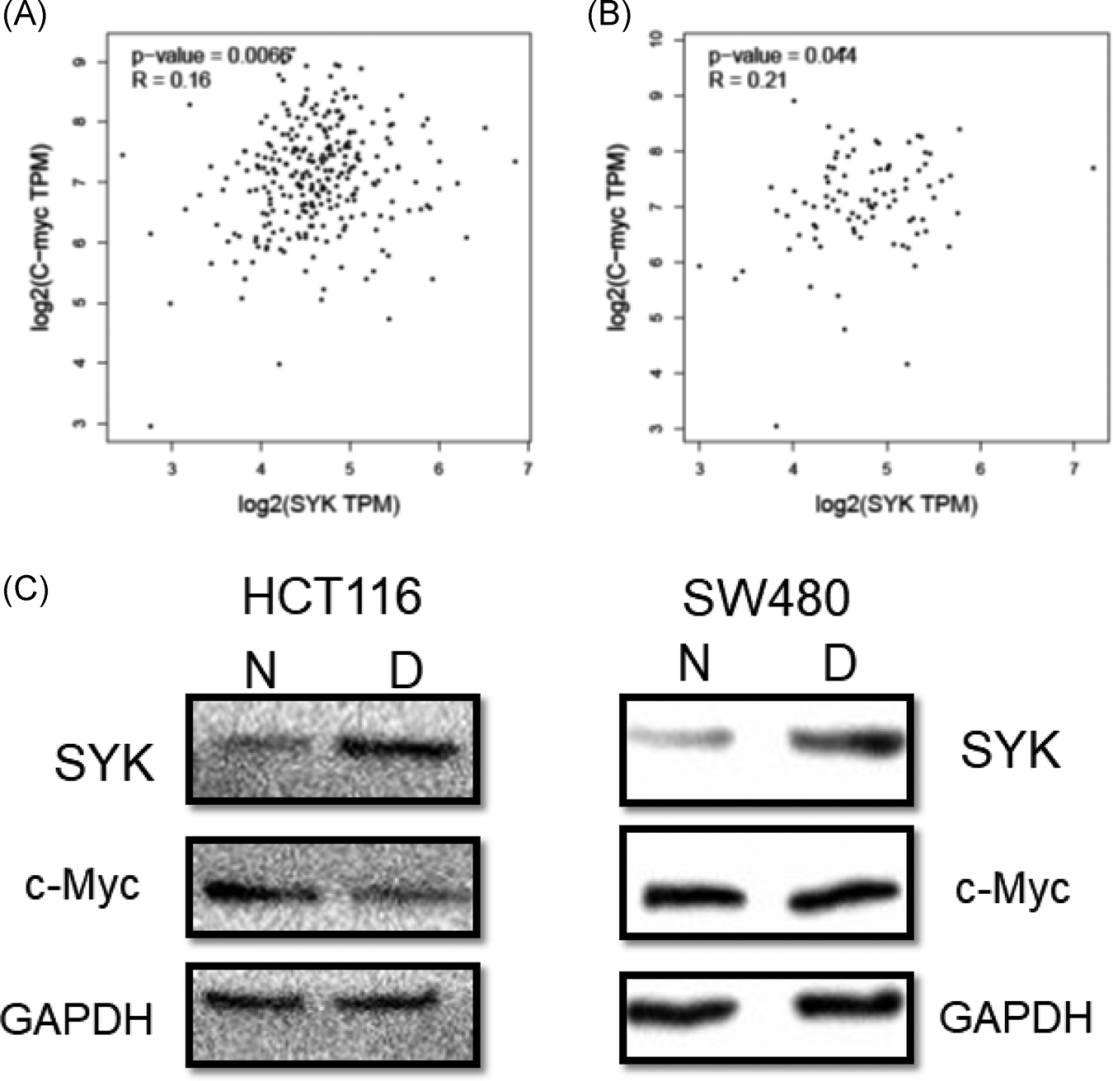
Correlation of Syk expression with c‐Myc. (A) Syk expression correlated with c‐Myc in colon adenocarcinoma based on GEPIA2. (B) Syk expression correlated with c‐Myc in rectal adenocarcinoma based on GEPIA2. (C) The expression of Syk negatively correlated with c‐Myc in HCT116 and SW480 using Western blot analysis. D, Decitabine; N, NC

Results from GEPIA2 uncovered that the expression of Syk was correlated with c‐Myc in colon adenocarcinoma (Figure 5A) and rectal adenocarcinoma (Figure 5B). Western blot analysis indicated that the expression of c‐Myc was downregulated after replenishment with decitabine in SW480 and HCT116 cells, while Syk expression was upregulated (Figure 5C). This finding implies that c‐Myc expression might be regulated by Syk.

## 4 DISCUSSION

Tumor metastasis is a multistep process in which tumor cells disseminate from their primary site to distant tissues or organs, forming a secondary tumor.^18^ CRC is one of the most lethal malignancies, the clinical outcome of metastatic CRC patients is particularly poor, and the 5‐year survival rate of metastatic colorectal patients was significantly worse compared to those without metastasis.^4^, ^19^ DNA methylation has recently been shown to be associated with tumorigenesis and metastasis of various cancers. The purpose of this study was to investigate the potential mechanism of Syk methylation regulating CRC metastasis.

In this study, we uncovered that the methylation level of Syk was altered in eight cancer categories, including colon adenocarcinoma in the UALCAN database. Previous studies have also reported that Syk was frequently methylated in CRC tissues and cell lines. Additionally, methylation of Syk is remarkably associated with histological grade, lymph node status, and TNM stage of CRC patients.^10^ Elsewhere, it has been found that the hypomethylation of Syk prohibited invasion of CRC cells and correlated with the prognosis of CRC patients, therefore, it was deemed as a predictive biomarker of CRC.^10^ Decitabine is an effective inhibitor of DNA methylation, which may restore the expression of hypermethylated genes. It has been applied in therapy for numerous cancers.^9^, ^20^, ^21^, ^22^ Herein, we demonstrate that replenishment with decitabine could restore the expression of Syk in SW480 and HCT116 cell lines. Similarly, recent studies have also found that decitabine could reactivate the expression of Syk in breast cancer and nasopharyngeal cancer cells.^23^, ^24^ The supplement of decitabine also affected the invasiveness of breast cancer cells.^23^ In addition, our results revealed that the migration and invasiveness ability of SW480 and HCT116 cells were affected by Syk expression. However, the underlying regulatory mechanism of Syk methylation on CRC cell invasion and migration remains largely unknown. Data from the TCGA database revealed that almost all progression of CRC is associated with the abnormalities of the targeted genes of c‐Myc.^25^ Here, we employed the GEPIA2 database and uncovered that the expression profile of Syk was correlated with c‐Myc. C‐Myc, like n‐myc and l‐myc, is one of the members of myc.^26^ Dysregulation of c‐Myc results in a variety of pathological conditions, such as uncontrolled cell proliferation and tumor metastasis.^27^, ^28^ Studies have elucidated that c‐Myc was upregulated in CRC, while in vivo experiments further demonstrated that prohibition of c‐Myc suppressed the tumorigenesis of CRC.^25^ Several studies also validate the correlation between the upregulation of c‐Myc and lymph node metastasis of CRC patients.^29^ We also found that the expression of c‐Myc was negatively correlated with Syk after replenishment with decitabine. Therefore, we believe that dysregulation of c‐Myc was regulated by Syk, which affected the metastasis of CRC.

Immunotherapy has become a crucial treatment strategy for patients with CRC.^30^ Therefore, we research the correlations between Syk expression with the contents of different immune cells. The results from CIBERSORT and TIMER revealed a strong correlation between Syk expression with immune features in colon adenocarcinoma. Results from CIBERSORT revealed that the expression profile of Syk was correlated with the contents of CD4^+^ memory resting T cell, M2 Macrophage, CD8^+^ T cells, activated NK cells, and resting mast cells in colon adenocarcinoma. Accumulating evidence indicates that NK cells participate in immune responses to cancer and can inhibit the spread of tumors.^31^ M2 macrophages play a role in anti‐inflammatory responses.^32^ Previous studies found that CD8^+^ T cells have antitumor immunity functions.^33^ Moreover, the methylation level of Syk has been reported to be correlated with immune‐stimulators such as CXCL12, ENTPD1, and TNFSF4. It was demonstrated that the combination of CXCL12 with CXCR4 and CXCR7 on tumor cells leads to antiapoptotic signals upregulated by Bcl‐2 and survivin, and affects the progression of gastrointestinal tumors.^34^ The soluble TNFSF4 in the blood is positively correlated with carbohydrate antigen (CA) 19‐9, carcinoembryonic antigen, C‐reactive protein, and soluble programmed cell death ligand‐1 in patients with CRC.^35^ These results indicate a strong correlation between Syk and immune features. However, we acknowledge the following limitations in our study. Further experiments are needed to validate the detailed mechanism of the regulation of CRC.

## 5 CONCLUSION

The findings of this study elucidate that decitabine can replenish Syk expression in CRC cells, thus affecting the migration and invasion of CRC. Moreover, the expression profile and methylation level of Syk were demonstrated to correlate with immune features in colon adenocarcinoma. However, the detailed regulated correlations of Syk and c‐Myc require to be confirmed in future studies.

## CONFLICT OF INTERESTS

The authors declare that there are no conflict of interests.

## AUTHOR CONTRIBUTIONS

*Project design*: Jun Huang. *Research and data collection*: Fanqin Bu, Xiaojian Zhu, and Kang Lin. *Statistics and data analysis*: Fanqin Bu and Sicheng Liu. *Writing*: Fanqin Bu and Jinfeng Zhu. All authors have read and approved the final version of the manuscript.
